# Multiplex Screening Assay for Identifying Cytotoxic CD8^+^ T Cell Epitopes

**DOI:** 10.3389/fimmu.2020.00400

**Published:** 2020-03-11

**Authors:** Chek Meng Poh, Jian Zheng, Rudragouda Channappanavar, Zi Wei Chang, Thi H. O. Nguyen, Laurent Rénia, Katherine Kedzierska, Stanley Perlman, Leo L. M. Poon

**Affiliations:** ^1^School of Public Health, Li Ka Shing Faculty of Medicine, The University of Hong Kong, Hong Kong, China; ^2^Department of Microbiology and Immunology, Carver College of Medicine, University of Iowa, Iowa City, IA, United States; ^3^Singapore Immunology Network, Agency of Science, Technology and Research, Singapore, Singapore; ^4^Department of Microbiology and Immunology, Peter Doherty Institute for Infection and Immunity, University of Melbourne, Melbourne, VIC, Australia

**Keywords:** influenza, *Plasmodium*, MERS, CD8 T cell, cytotoxicity, multiplex assay

## Abstract

The cytotoxicity of epitope-specific CD8^+^ T cells is usually measured indirectly through IFNγ production. Existing assays that directly measure this activity are limited mainly to measurements of up to two specificities in a single reaction. Here, we develop a multiplex cytotoxicity assay that allows direct, simultaneous measurement of up to 23 different specificities of CD8^+^ T cells in a single reaction. This can greatly reduce the amount of starting clinical materials for a systematic screening of CD8^+^ T cell epitopes. In addition, this greatly enhanced capacity enables the incorporation of irrelevant epitopes for determining the non-specific killing activity of CD8^+^ T cells, thereby allowing to measure the actual epitope-specific cytotoxicity activities. This technique is shown to be useful to study both human and mouse CD8^+^ T cells. Besides, our results from human PBMCs and three independent infectious animal models (MERS, influenza and malaria) further reveal that IFNγ expression by epitope-specific CD8^+^ T cells does not always correlate with their cell-killing potential, highlighting the need for using cytotoxicity assays in specific contexts (e.g., evaluating vaccine candidates). Overall, our approach opens up new possibilities for comprehensive analyses of CD8^+^ T cell cytotoxicity in a practical manner.

## Introduction

After priming and activation by antigen-presenting cells in secondary lymphoid organs, epitope-specific CD8^+^ T cells expand in numbers, migrate to sites of infection, and kill infected cells (1). CD8^+^ T cells also form part of the memory response. They can rapidly expand in number and eliminate pathogens after secondary challenge. Also, CD8^+^ T cells can recognize tumor antigens presented by cancer cells and eradicate them before they metastasize (2). The cytotoxicity of CD8^+^ T cells requires direct contact with target cells, although the capability of each CD8^+^ T cell to eliminate target cells remains uncertain (3, 4). Regardless, these cell-to-cell contacts enable CD8^+^ T cells to release cytotoxic granules and death ligands, thereby killing the target cells (5).

Interferon-gamma (IFNγ) is one of the critical cytokines for establishing effective immune responses. In the context of T cells, this cytokine helps to drive the polarization of CD4^+^ T cell response to T_H_1, which directs the overall T cell response toward cell-mediated immunity. In CD8^+^ T cells, activated subsets start to produce IFNγ rapidly, with the dominant epitope-specific CD8^+^ T cells expanding preferentially over subdominant ones (6, 7). IFNγ production by activated CD8^+^ T cells also promotes T_H_1 polarization (8), enhances cell motility and cytotoxicity (9), and exerts antiviral immunity in a wide variety of cells through IFNγ receptor signaling (10). IFNγ also plays a vital role in regulating the contraction of effector CD8^+^ T cells after the clearance of primary infection (11, 12). Despite its multiple functions, IFNγ produced by activated epitope-specific CD8^+^ T cells is widely used as an indirect marker to demonstrate the functional efficacy of epitope-specific CD8^+^ T cells. For example, as assays for measuring IFNγ produced by CD8^+^ T cells can be highly robust (e.g., Intracellular Cytokine Staining, ICS), IFNγ is often used as a surrogate marker in screening assays for identifying potential cytotoxic CD8^+^ T cell epitopes. However, one should note that the relationship between these two parameters is not necessarily correlated.

The cytotoxicity of CD8^+^ T cells against their cognate targets can be measured directly, which was initially assayed through radioactive labeling of target cells, mostly using chromium-51 (13). Fluorescence-based methods have since supplanted the radioactive labeling methods due to their higher sensitivity and better safety profile (14). However, these assays are typically used in a single target format, allowing measurements of cytotoxic potential of one or two CD8^+^ T cell attributes. This approach is not highly robust, which limits its potential use in large-scale screening studies. When there is a need to test many CD8^+^ T cells epitopes, the amount of starting materials (mice or human PBMCs) that are needed will inevitably increase substantially, which may pose difficulties for conducting comprehensive analyses.

In this study, we demonstrated the feasibility of measuring cytotoxicity activities of up to twenty-four specificities using just three fluorescent dyes in a single reaction, thereby increasing the multiplexity and capability to screen multiple CD8^+^ T cell epitopes simultaneously. We used CD8^+^ T cells from human PBMCs to analyze previously known HLA-A11^*^01-restricted epitopes of influenza A viruses *in vitro*, showing that only some of these epitopes can elicit a robust cytotoxic response. In mouse models, we demonstrated that this method allows for screening of CD8^+^ T cells harvested from different anatomical sites and that our approach allows accurate measurement of cell killing that is attributable to cognate CD8^+^ T cells. We further demonstrated that IFNγ expression by epitope-specific CD8^+^ T cells does not necessarily correlate with its cytotoxic potential. Our results highlight that this multiplex assay has potential uses in identifying potent cytotoxic CD8^+^ T cell epitopes. Besides, epitopes that have differential effects on the IFNγ production and cytotoxic activity of CD8^+^ T cells can be identified by this approach in a rapid manner.

## Materials and Methods

### Peptides

Peptides of >90% purity were purchased from GenScript (Piscataway, NJ), reconstituted at 10 mg/mL with dimethyl sulphoxide (DMSO), and stored at−20°C.

### Study Subjects

All healthy donors and patients provided written, informed consents to participate in the study. Ethics approvals were obtained from the Human Research Ethics Committee of the University of Hong Kong and Monash Health (HREC/15/MonH/64), Royal Melbourne Hospital (local reference number: 2016/196). Blood samples from the subjects were screened for blood-borne pathogens and sent for Human Leukocyte Antigen (HLA) typing. Patient blood samples were collected in sodium heparin tubes and PBMCs isolated as previously described (15). Briefly, blood was diluted 1:1 (v/v) with PBS, overlaid on Histopaque-1077 (Sigma-Aldrich, St. Louis, MO) and centrifuged at 400 g for 30 min without brake. Isolated PBMCs were washed with PBS and resuspended in FBS+10% DMSO for liquid nitrogen storage.

### Mice

Six-to eight-week-old BALB/cJ, C57BL/6J, and human dipeptidyl peptidase-4 knock-in (hDPP4-KI) mice on C57BL/6J background (16) were used in these experiments. Mice were bred under specific-pathogen-free conditions. All animal experiments and procedures were approved by the following: (1) Committee on the Use of Live Animals in Teaching and Research, The University of Hong Kong (Guidelines for the use of experimental animals); (2) Institutional Animal Care and Use Committee, Agency of Science, Technology and Research, Singapore (Guidelines of the Agri-Food and Veterinary Authority) or (3) Institutional Animal Care and Use Committee, University of Iowa (Guidelines: National Advisory Committee for Laboratory Animal Research). Animal experiments with Middle East respiratory syndrome coronavirus (MERS-CoV) were conducted in the University of Iowa animal biosafety level 3 (ABSL-3) facilities.

### Viruses and Parasites

A vaccinia virus carrying H5N1 NP, HA, NA, M1, M2 genes, and human IL-15 previously reported by us was used as an experimental vaccine (17). Mice were anesthetized and given 1 x 10^7^ PFU of this vaccine through the intranasal route twice at 3 weeks apart. Three weeks after the second vaccination, mice were challenged with 10 MLD_50_ H1N1 (A/Puerto Rico/8/34) through the intranasal route. Weight loss and survival of mice were followed for up to 2 weeks. *Plasmodium berghei* ANKA clone 15Cy1 (PbA) (18) and *Plasmodium berghei* NK65 (PbNK65) (19) parasites were passaged in C57BL/6J mice, and infected erythrocytes were resuspended in Alsever's solution and stored in liquid nitrogen. To infect mice, 1 × 10^6^ infected erythrocytes were injected through the intraperitoneal route. Parasitaemia was monitored by flow cytometry (20). All MERS-CoV experiments were carried out in the University of Iowa ABSL-3 facility. Mice were infected with 1 × 10^5^ PFU of human isolate of MERS-CoV (MERS-CoV-EMC) and these mice were challenged after 4 weeks with 2 × 10^3^ PFU of a mouse-adapted strain of MERS-CoV.

### Multiplex *in vivo* Cytotoxicity Assay With Donor Splenocytes

Spleens from naïve mice were dissociated using a 70 μm cell strainer with a syringe piston to release splenocytes in RPMI complete medium, supplemented with 10% fetal bovine serum (FBS) and 100 U/mL penicillin-streptomycin (ThermoFisher Scientific, Waltham, MA). Splenocytes were resuspended with ACK lysis buffer (155 mM NH_4_Cl, 10 mM KHCO_3_, 0.2 mM EDTA; all chemicals from Sigma-Aldrich) for at least a minute before washing with RPMI complete medium. The splenocytes were split into up to 24 groups and pulsed with relevant peptides at a final concentration of 10 mg/mL. Treated cell were labeled with unique combinations of CellTracker CMFDA, CMTMR, and Deep Red dyes (ThermoFisher Scientific; Table S1) and were then washed with RPMI complete media. Equal numbers of labeled cells from each group were combined and transferred into recipient mice at a total volume of 30 μL and 200 μL PBS for intranasal and retro-orbital routes, respectively. After 16–20 h, recipient mice were sacrificed to harvest donor splenocytes and bronchoalveolar lavage, which were labeled with Live/Dead Fixable Violet stain (ThermoFisher Scientific) before acquisition by flow cytometry.

### Multiplex *in vitro* Cytotoxicity Assay With Human PBMCs

Thawed PBMCs were washed twice with RPMI complete medium, resuspended in 10 mL fresh medium in 50 mL Falcon tube and left to recover at 37°C, 5% CO_2_ overnight at about 5° horizontal tilt with loose cap (21). After recovery, cells were split into two groups: one group was treated with CD8^+^ T cell isolation kit and the other group treated with CD8^+^ Nanobeads for depletion (Biolegend, San Diego, CA). The target cells obtained from the negative fraction from the latter group were split into groups for peptide pulsing and dye labeling as described earlier. Equal numbers of cells from each group were then combined together and split into two groups: one group to be mixed with the isolated CD8^+^ T cells and the other group without. Cells were seeded in a 96-well flat-bottom plate and incubated at 37°C, 5% CO_2_ overnight. The next day, cells were labeled with Live/DEAD Fixable Near IR stain (ThermoFisher Scientific) before acquisition using a flow cytometer.

### IFNγ-Intracellular Cytokine Staining (IFNγ-ICS)

Splenocytes from mice at up to 5 × 10^6^ cells were seeded together with 5 mg/mL mouse IL-2 (Biolegend), 1 μL BD GolgiPlug (Becton Dickinson, Franklin Lakes, NJ) or Brefeldin A (ThermoFisher Scientific) and 10 μg peptide in 96-well tissue culture plates, followed by incubation at 37°C, 5% CO_2_ for 5 h. For *Plasmodium* studies, the mouse IL-2 addition was omitted. They were then stained with Zombie Aqua Fixable Viability kit (Biolegend) for 30 min, washed and followed by antibody stainings for 30 min, selected from the following panel: purified CD16/32 (clone 93); CD3 PE/Dazzle 594 (clone 17A2); CD3ε Brilliant Violet 421 (clone 145-2C11); CD4 APC/Cy7 (clone GK1.5); CD8a PerCP/Cy5.5 (clone 53-6.7); CD8b PerCP/Cy5.5 (clone YTS 156.7.7, all from Biolegend). After washing, cells are fixed in 4% formaldehyde, permeabilized with 0.1% saponin (Sigma-Aldrich) or per buffer (BD Bioscience), followed by staining with IFNγ FITC (clone XMG1.2, Biolegend/eBioscience).

### Specific Lysis Calculation

The formula for the calculation of cytotoxicity of antigen-specific CD8^+^ T cells are as follows:

(1) $$\begin{array}{l} \left[1-\frac{{\left(\frac{DMSO\;group}{Peptide-pulsed\;group}\right)}_{Naive}}{{\left(\frac{DMSO\;group}{Peptide-pulsed\;group}\right)}_{Experimental\;group}}\right]\times \;100\% \end{array}$$

### Statistical Analysis

Mann–Whitney *U*-test was used to analyse for statistical significance between two groups. For comparisons between more than two groups, one-way analysis of variance (ANOVA) was used if sample data follow a normal distribution. Otherwise, Kruskal–Wallis test and Dunn's post-test were used. Dunnett's or Dunn's multiple comparisons were performed with the irrelevant control (CSP280, OVA, MOG, or Ebo580-588) designated as the control group. Flow cytometric analysis was carried out with FlowJo (TreeStar, Ashland, OR) and calculations were performed with Prism 6 (GraphPad, La Jolla, CA).

## Results

### Expansion of Target Groups to Increase Multiplexity

**Figure 1:**
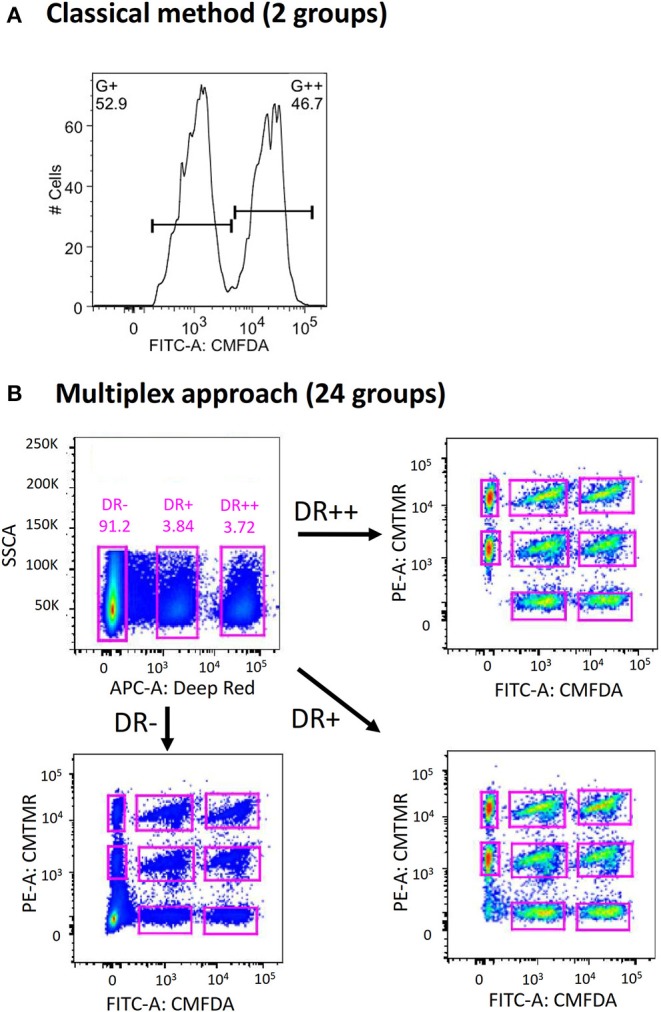
Expansion of conventional *in vivo* cytotoxicity assay to increase multiplexity. **(A)** In conventional *in vivo* cytotoxicity assay, two groups of donor splenocytes (one of which is pulsed with peptide of interest) were labeled differentially with a cell tracking dye (such as CellTracker CMFDA) and transferred into recipient mice intravenously. These can be distinguished from each other during flow cytometric analysis. **(B)** By incorporating two more CellTracker dyes (CMTMR and Deep Red) and combinatorial labeling, 24 distinct groups of donor splenocytes can be created, adoptive transferred, harvested and analyzed.

In contrast to the conventional method of using a single cell labeling dye (e.g., CMFDA) to create two distinct target populations during flow acquisition (Figure 1A), we first explored the possibility of combining three different labeling dyes to exponentially increase the number of target populations that can be distinguished (Figure 1B). This strategy was inspired by the use of multicolor-coded MHC multimers to test different antigen-specific T-cell responses in a single sample (22). By modifying the concentrations of each dye, up to 24 different groups of donor cells can be created (Table S1). When equal numbers of cells from each group were combined and transferred into recipient mice through the intravenous route, all 24 different groups of donor cells could be identified by flow cytometry (Figure 1B). The clear demarcation of each donor group raises the possibility of directly measuring the cytotoxicity of multiple CD8^+^ T cell specificities simultaneously in a single mouse (see below). This possibility is in sharp contrast to the conventional method, which requires one mouse for testing a single CD8^+^ T cell epitope.

### Multiplex *in vivo* Cytotoxicity Assay Allows Simultaneous *in vivo* Measurement of Different CD8^+^ T Cell Specificities in Different Compartments

**Figure 2:**
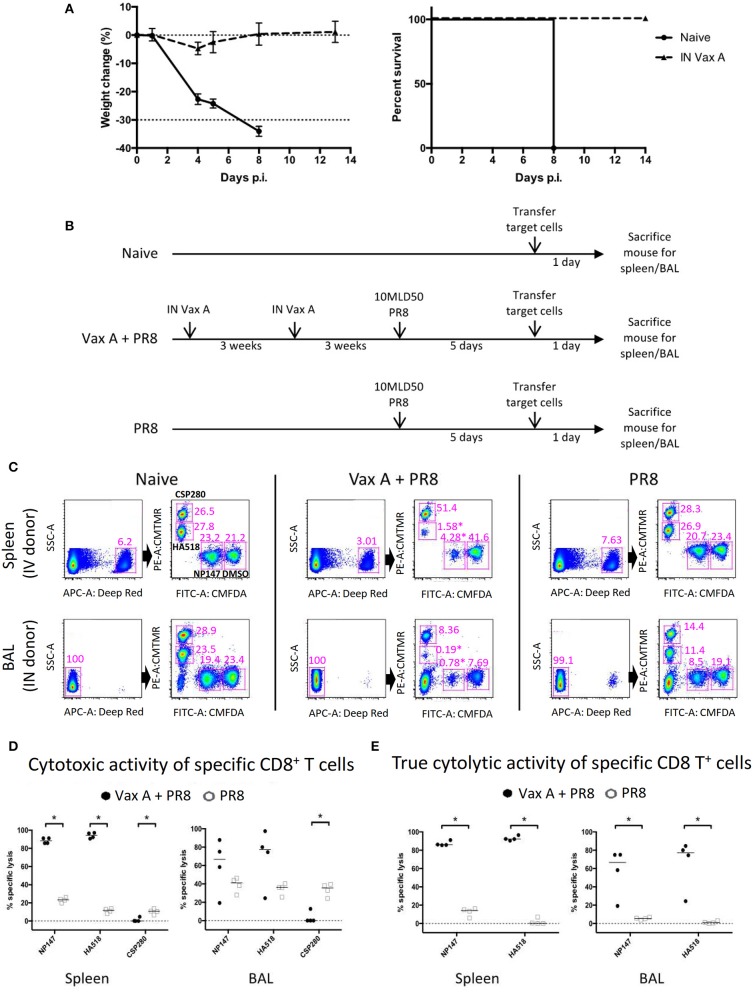
Using multiplex *in vivo* cytotoxicity assay to simultaneously measure multiple CD8^+^ T cell specificities in different anatomical locations. **(A)** Mice were vaccinated twice with 1 × 10^7^ PFU Vax A through the intranasal route, then challenged with 10 MLD_50_ PR8 IAV through the intranasal route. The weight change and survival rate over a period of 2 weeks are shown. **(B)** The schematics describe the experimental workflow for multiplex *in vivo* cytolytic assay in naïve, vaccinated and unvaccinated mice receiving lethal influenza challenge. **(C)** Naïve donor splenocytes were processed as target cells according to Table S2, then inoculated into recipient mice and re-harvested the next day to analyze remaining target cells. Representative flow plots of mice from different experimental groups were shown, depicting the gating strategies to identify corresponding donor cell groups. Corresponding positions for target cells treated with CSP280, HA518, NP147, and DMSO are highlighted in the top right panel for naïve mice as examples. **(D–E)** The analysis of cytolytic activity of specific CD8^+^ T cells, before **(D)** and after **(E)** normalization, were shown. *p < 0.05, Mann–Whitney *U*–test.

We previously developed a vaccinia-based influenza A virus (IAV) vaccine (henceforth called Vax A) that can express five H5N1 IAV genes and human interleukin-15 gene in mice (17). This experimental vaccine is known to induce robust CD8^+^ responses in mice. Mice vaccinated twice with Vax A via intranasal route could be fully protected by a lethal heterologous influenza virus challenge (A/PR/8/34; 10 MLD_50_), whereas all unvaccinated mice succumbed to the challenge (Figure 2A).

We used this animal model to evaluate our multiplex *in vivo* cytotoxicity assay. We divided mice into three groups, and they were subjected to different treatments (Figure 2B. Naïve: no treatment; Vax A + PR8: vaccination followed by a virus challenge; PR8: virus challenge only). At day 5 post-challenge, we prepared donor splenocytes from unrelated naïve mice and divided them into eight groups. Each group of donor cells were pulsed and labeled with a specific combination of peptide and dye (Table S2). The treated donor cells were then transferred into these mouse groups via intravenous or intranasal routes (Figure 2C, upper and lower panel), and they were recovered from these recipient mice the following day. As shown in Figure 2C, we could identify these labeled cells and distinguish them from each other in all experimental conditions. Furthermore, donor cells pulsed with influenza epitopes NP147 (CMFDA+) and HA518 (CMTMR+) experienced high levels of cytolysis in vaccinated mice upon challenge with influenza (Figure 2C, highlighted by ^*^ in the middle panels, and Figure 2D), but not in unvaccinated mice. However, the CSP280 (CMTMR++) control group experienced significant cytolysis in both the spleen and bronchoalveolar lavage of the unvaccinated mice, with this effect more pronounced in latter tissue compartment (Figure 2D, left vs. right panels). This observation suggested that the inflammatory microenvironment contributed to substantial non-specific killing in these unprotected mice. The proinflammatory milieu is known to induce Fas receptor expression in different cells (23–25), and others have shown that bystander cell lysis by activated CD8^+^ T cells occurs in a TNFα- or FasL-dependent manner (26–28). Thus, we believe that the non-specific cytolysis by activated CD8^+^ T cells in our model could occur similarly. We also believe that the bystander killing of target cells due to dysregulated inflammation resulting from IAV infection can also extend to cells labeled with IAV epitopes. Thus, the inclusion of irrelevant epitopes in this multiplex assay provides a unique opportunity to quantitate this bystander killing effect. Consequently, we used CSP280 data points from individual mice to normalize against the rest of the corresponding data points to obtain Figure 2E. These normalized data clearly showed that CD8-mediated cytolysis against influenza epitopes are also elevated in the lung mucosa of vaccinated mice.

### Analyzing Cytotoxicity of Multiple Epitope-Specific Human CD8^+^ T Cells Simultaneously *in vitro*

Next, we tested the potential of this multiplex approach using CD8^+^ T cells harvested from human PBMCs. We obtained frozen PBMC samples from HLA-A^*^11:01-positive healthy individuals and influenza-infected patients to purify CD8^+^ T cells. We also harvested homologous non-CD8^+^ cells from the corresponding samples and use them as target cells in the assay. These target cells were divided into groups and pulsed with different HLA-A^*^11:01-restricted influenza virus epitopes (Table S3). These epitopes are experimentally known to induce influenza-specific CD8^+^ T cell responses in HLA-A^*^11:01-positive individuals (Influenza Research Database and Immune Epitope Database). Peptide-pulsed target groups were differentially labeled with unique combinations of fluorescent markers, pooled together and split equally into two groups, with one group incubated together with homologous CD8^+^ T cells and the other group without. The latter group serves as the naïve population, which is essential for the calculation of epitope-specific CD8^+^ T cell cytotoxicity.

**Figure 3:**
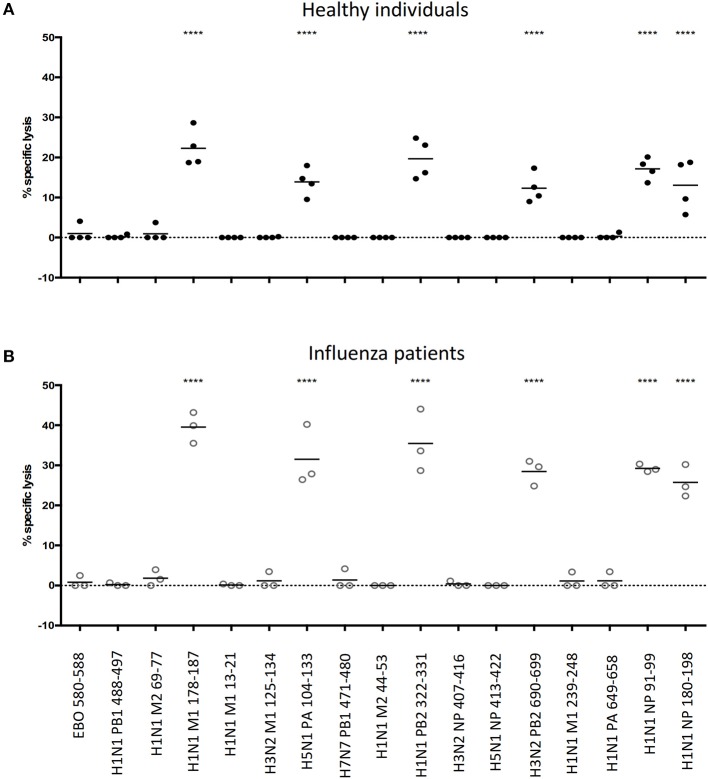
Feasibility of performing multiple *in vitro* cytotoxicity assay in a single well. Frozen PBMCs from **(A)** healthy and **(B)** influenza-infected HLA-A*11:01 individuals were rested in a 37°C/5% CO_2_ incubator overnight. Non-CD8^+^ T cells (target cells) were isolated from CD8^+^ T cells by MACs, split into equal groups and pulsed with corresponding peptides (including one additional group that is incubated with DMSO; peptide sequences are shown in Table S3). Pulsed target cells are combinatorically labeled with cell labeling dyes (CellTrace Violet, CellTrace Yellow, CellTracker CMFDA and CellTracker Deep Red), mixed in equal numbers and split into two groups: one group incubated with CD8^+^ T cells and the other group without. Cells are incubated at 37°C/5% CO_2_ overnight and analyzed the next day by flow cytometry to calculate the specific lysis of target cells by cognate CD8^+^ T cells. ****p < 0.0001, One-way ANOVA with Holm-Sidak's multiple comparison test against Ebo 580-588 (control).

Of sixteen tested IAV epitopes, six (H1N1 M1 178-187, H5N1 PA 104-113, H1N1 PB2 322-331, H3N2 PB2 690-699, H1N1 NP 91-99, and H1N1 NP 188-198) could evoke significant levels of cytotoxicity from their cognate CD8^+^ T cells, both in healthy individuals (Figure 3A) and in hospitalized patients with acute influenza (Figure 3B). For these six cytotoxic CD8+ T cell epitopes, infected individuals consistently elicited stronger responses than the healthy individuals did (One-way ANOVA with Holm-Sidak's post-comparison; *p* < 0.0001). Hence, apart from identifying multiple epitope-specific CD8^+^ T cells that elicited cytotoxicity toward their targets simultaneously, the results of the multiplex *in vitro* cytotoxicity assay correlated with the immune status of individuals, i.e., the rapid proliferation of influenza-specific CD8^+^ T cells in infected individuals led to elevated levels of cytotoxic activities.

Ebola does not circulate in the region where our PBMC donors reside. We reasoned that HLA-A^*^11:01-restricted Ebola virus epitope, EBO 580-588 (29) could serve as an appropriate irrelevant epitope to demonstrate the specificity of our multiplex cytotoxicity assay, as well as setting a baseline for comparison. As expected, no cytotoxic activity against Ebo 580-588 could be detected, proving the specificity of this multiplex assay for testing human CD8^+^ T cells from the blood.

### Cytotoxicity of Epitope-Specific CD8^+^ T Cells Can Differ From Corresponding IFNγ Production

All influenza virus epitopes used in the above human study were previously shown to stimulate CD8^+^ cell responses in various immune assays (Table S3). We were surprised to find that only a minority of these epitopes can elicit cytotoxic killing from their cognate CD8^+^ T cells. As HLA-typed PBMC samples were highly limited, it was difficult for us to study this phenomenon comprehensively. Therefore, we used different animal models to test whether cytotoxicity of epitope-specific CD8+ T cells can differ from corresponding IFNγ production.

**Figure 4:**
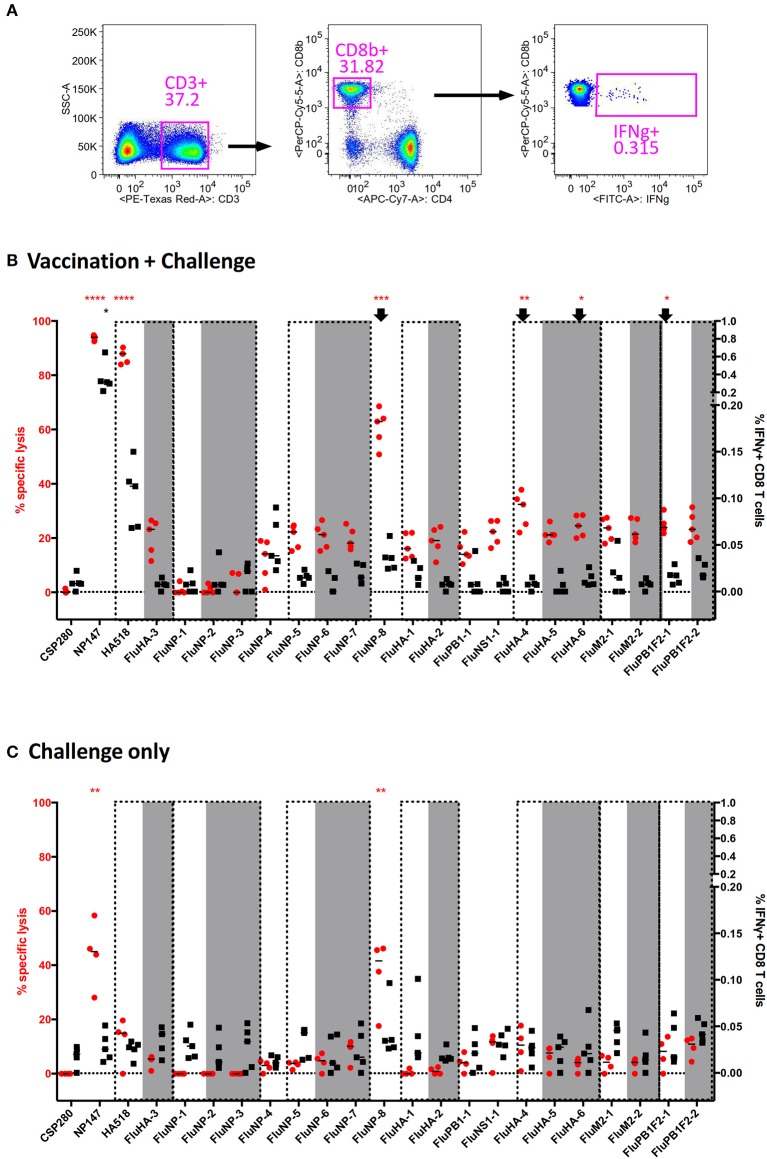
Discrepancy between direct and indirect measurements of CD8^+^ T cell effector functions in an influenza mouse model. Splenocytes from vaccinated mice challenged by a lethal PR8 infection were used to measure epitope-specific CD8 T cell effector functions through direct (*in vivo* cytotoxicity) and indirect (IFNγ-ICS) assays. Twenty-three different peptides were employed for the screening (Table S4) to fully utilize the capability of our multiplex assay. **(A)** The representative gating strategy for IFNγ-ICS assay is shown. **(B–C)** The results for vaccinated **(B)** and unvaccinated **(C)** mice following challenge are shown. Epitopes grouped within dashed boxes are homologs of each other, with those in gray originating from H3N2 or H5N1. ****p < 0.0001, ***p < 0.001, **p < 0.01, *p < 0.05, Kruskal-Wallis test with Dunn's post-test against irrelevant control (CSP280). Red and black asterisks refer to corresponding data from multiplex *in vivo* and IFNγ-ICS assays respectively.

We first used the vaccinated mouse model described above to check for this phenomenon. We expanded the number of epitopes to be tested by searching through the Influenza Research Database for H2-K^d^-binding PR8 H1N1 epitopes and their variants found in H3 or H5 subtype (if available). We selected a total of 24 epitopes for a 3-color combination screening (Table S4). We also chose to perform the multiplex *in vivo* cytotoxicity assay and IFNγ-ICS assay (Figure 4A) in separate experiments in order to avoid issues of spillover signals from brightly labeled donor splenocytes. Both results were then overlaid onto a single graph to allow for better visual comparison (Figure 4B; non-H1N1 viral epitopes are highlighted in gray and variant epitopes are in the same dotted box).

In vaccinated mice subjected to a lethal PR8 challenge, the immunodominant epitopes NP147 and HA518 consistently produced robust responses in both assays (Figure 4B, Left 2 and 3). However, the following epitopes did not give concordant results: FluNP-8, FluHA-4, FluHA-6, and FluPB1F2-1 (Figure 4B, arrows). Especially for FluNP-8, the peptide elicited a very significant positive response in cytotoxicity assays but not in assays measuring IFNγ expression. Interestingly, with the exception of FluHA-3, IAV variant epitopes that were present in H3N2 or H5N1 subtypes elicited similar cytotoxicity and IFNγ expression by CD8^+^ T cells as the corresponding homologs present in PR8, suggesting that PR8-specific CD8^+^ T cells may tolerate small changes in the epitope sequence, enabling them to target cells infected by other IAV subtypes. In unvaccinated mice, cytotoxicity against NP147 and FluNP-8 were significantly elevated upon lethal PR8 challenge, albeit not at levels seen in vaccinated mice (Figure 4C). More importantly, intracellular IFNγ staining did not show any significant difference between any of the tested peptides.

**Figure 5:**
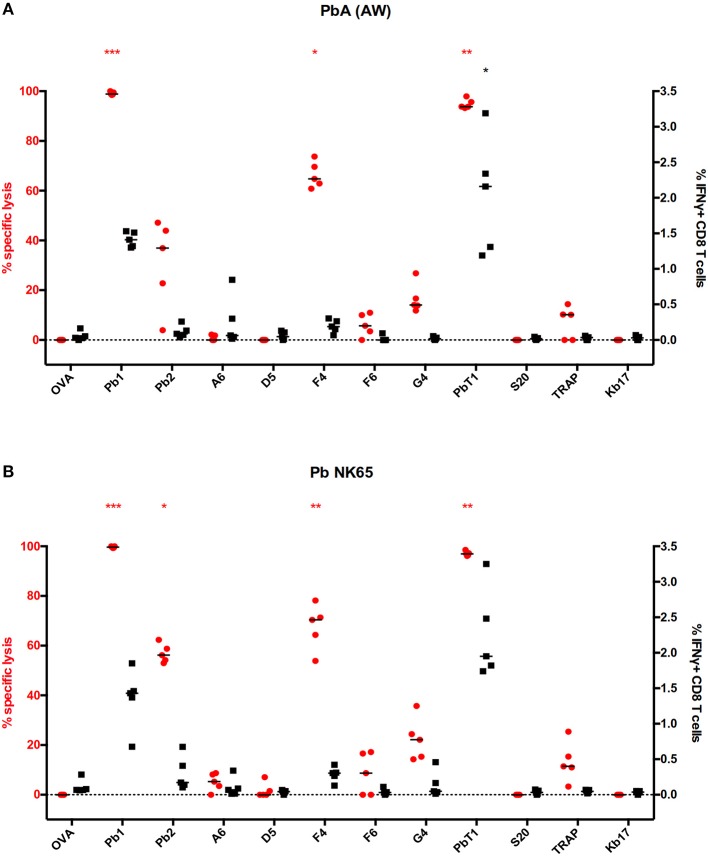
Discrepancy between direct and indirect measurements of CD8 effector functions in a malaria mouse model. **(A–B)** Splenocytes from C57BL/6J mice infected with **(A)** *Plasmodium berghei* ANKA (PbA) or **(B)** *Plasmodium berghei* NK65 were used to measure specific CD8 T cell effector functions through multiplex *in vivo* cytotoxicity and IFNγ-ICS assays. Eleven malaria peptides (Table S3) that were published in the literature were employed in the screens, with the results as shown. ***p < 0.001, **p < 0.01, *p < 0.05, Kruskal–Wallis test with Dunn's post-test against irrelevant control (OVA). Red and black asterisks refer to corresponding data from multiplex *in vivo* and IFNγ-ICS assays respectively.

In the above mouse work, we observed apparent discrepancies between IFNγ production, a common marker of T cell activation, and actual cytotoxicity in some IAV-specific CD8^+^ T cells. We wondered if we could observe this phenomenon in other disease models. To answer this, we used the pathogenic malaria model for evaluation. The malaria parasite *Plasmodium berghei* ANKA (PbA) causes a severe neurological complication termed experimental cerebral malaria in susceptible C57BL/6J mice. At 6 days post-infection, mice infected with PbA were subjected to both assays with the eleven previously known H-2^b^ malaria epitopes (Table S5). In PbA-infected mice, Pb1-, F4-, and PbT1-specific CD8^+^ T cells displayed significantly elevated killing activities. Interestingly, only Pb1- and PbT1-specific CD8^+^ T cells, but not those specific for the F4 epitope, registered significant increases in IFNγ production (Figure 5A). A similar result also occurs when a different parasite strain, PbNK65, was used to infect mice (Figure 5B).

**Figure 6:**
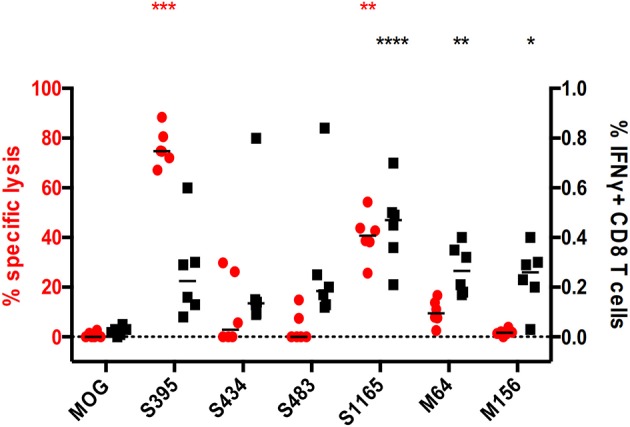
Discrepancy between direct and indirect measurements of CD8^+^ T cell effector functions in a Middle East Respiratory Syndrome (MERS) mouse model. The hDPP4-KI C57BL/6J mice were infected with 1 × 10^5^ MERS-CoV-EMC via intranasal route. After 4 weeks, these mice were challenged with 2 × 10^3^ PFU of mouse-adapted strain of MERS-CoV via intranasal route. Spleens from these mice were harvested and stimulated with the corresponding peptides in the presence of Brefeldin A. IFNγ expression from CD8 T cells in the splenocytes were quantified (black). In parallel, splenocytes from healthy donor mice were used for multiplex *in vivo* cytotoxicity assay with the corresponding peptides and transferred into transduced/challenged recipients at day 5 post-infection. The recipient splenocytes were harvested and analyzed by flow cytometry (red). *p < 0.05, **p < 0.01, ***p < 0.001, ****p < 0.0001, Kruskal–Wallis test with Dunn's post-test against irrelevant control (OVA). Red and black asterisks refer to corresponding data from multiplex *in vivo* and IFNγ-ICS assays respectively.

Additionally, we investigated this phenomenon in a mouse model of MERS-CoV infection (16). In this model, hDPP4-KI mice were infected with MERS-CoV-EMC. After 4 weeks, these mice were challenged with a 4LD_50_ (2000 PFU) mouse-adapted strain of MERS-CoV. At the peak of the T cell response, splenocytes were harvested and assayed for epitope-specific CD8^+^ T cell activity, with the epitopes shown in Table S6, by IFNγ expression and *in vivo* cytotoxicity assays. As shown in Figure 6, CD8^+^ T cells recognizing all the tested CD8^+^ T cell epitopes show elevated IFNγ levels in challenged mice, but this was not corroborated in cytotoxic assays. When comparing S395- and M156-specific CD8^+^ T cell activities, the former showed potent cytotoxicity while the latter had no cytotoxicity, despite both of them having comparable levels of IFNγ expression (Figure 6).

## Discussion

In this study, we expanded the conventional *in vivo* cytotoxicity assay from two parameters to twenty-four parameters. This functional assay allows the direct assessment of multiple CD8^+^ T cell specificities simultaneously in a single mouse or human PBMC sample, leading us to the finding that not all epitope-specific CD8^+^ T cells that express IFNγ exhibit concomitant direct cytotoxicity. Importantly, we demonstrate that this phenomenon is not limited to three different pathogen mouse models: IAV, malaria and MERS-CoV, but it also appears on human PBMC screening against experimentally known IAV CD8^+^ epitopes. These observations raise concerns about T cell studies in applications such as validating epitope candidates for vaccine studies. Currently, IFNγ-ICS remains widely used for such a purpose, with those eliciting IFNγ production regarded as responding positively to a pathogen insult, leading them to be selected as potential targets of vaccine design. However, this may not mean the T cells induced by this kind of epitope-based vaccines can always control the disease, as has been shown in a simian immunodeficiency virus vaccination/challenge study (30), and a human IAV trial (31). Although no cytotoxicity data were obtained in those studies, we argue that the possible discrepancies between IFNγ production and actual cytotoxic potential of the epitope-specific CD8^+^ T cells may be one of the causes. In this respect, we believe our multiplex cytotoxicity assay can further validate the epitope candidates in terms of the cytotoxicity potential of the cognate CD8^+^ T cells. In particular, our approach can be a robust tool for revealing a more comprehensive picture of the protective role of CD8^+^ T cells stimulated by T cell-based vaccines. Furthermore, the multiplex cytotoxicity assay is also useful for evaluating CD8^+^ T cell cytotoxicity responses in T cell therapies such as those involving chimeric antigen receptor (CAR)-T cells or cancer vaccines.

The multiplex cytotoxicity assay described here produces results that are comparable to that when the conventional single-plex cytotoxicity assay was performed instead (data not shown), which offers several advantages. Firstly, the number of recipient mice needed in a given experimental setup can be reduced, resulting in lower costs, better compliance with animal ethics (the 3R rule), and data that are more reproducible across multiple epitopes. Secondly, CD8^+^ T cell killing activities in different compartments can be analyzed simultaneously, which will provide data useful for evaluating the efficacy of a vaccine in inducing both systemic and mucosal adaptive immunity. Thirdly, the expansion of parameters available for screening allows one to include one or more irrelevant epitopes in the cytotoxicity assay. By definition, irrelevant epitopes should not elicit destruction by CD8^+^ T cells. However, it has been shown that uninfected cells also undergo apoptosis and necrosis during IAV infection (32). Hence, we argue that such non-specific killing can apply to intended target cells, and the destruction of irrelevant target cells acts as a control to measure non-specific killing activity, permitting the calculation of actual CD8^+^ T cell-mediated cytotoxicity levels.

The expression of IFNγ in epitope-specific CD8^+^ T cells has been widely used to measure cell activation and hence represents a marker to evaluate the efficacy of CD8^+^ T cell epitopes. This expression and other commonly used maker staining methods (e.g., tetramer staining) are indirect methods of assessing T cell functionality. One would expect that CD8^+^ T cells that upregulate IFNγ production should be cytolytic against its cognate targets. However, in an HIV setting, single-cell analysis of individual HIV-specific CD8^+^ T cells *in vitro* revealed discordance between IFNγ expression and cytolytic potential (33). In three different mouse models of infection tested in our study, we showed that such discrepancies also occur in some epitope-specific CD8^+^ T cells. In most cases, the discrepancies exist in specific CD8^+^ T cells that were IFNγ-negative but possess vigorous cytolytic activity. However, this can also happen vice versa. Continuous IFNγ signaling by activated epitope-specific CD8^+^ T cells was shown to lead to their reduction of cytolytic potential (34), suggesting that these cells may downregulate IFNγ production in order to maintain their cytolytic potential. On the other hand, the finding of substantial IFNγ production by CD8^+^ T cells that possess low cytotoxic potential, such as the M64- and M156-specific CD8^+^ T cells in our MERS-CoV-infected mice, was also unusual. Similar phenomena could also be found in humans (35), but the mechanism behind this is yet to be determined.

The mechanisms regulating the IFNγ production and cytolytic potential of epitope-specific CD8^+^ T cells may be highly complex, and future research on this topic is needed. Nevertheless, our results point out the disadvantage of relying solely on IFNγ production as a marker to evaluate CD8^+^ T cell activation, particularly in screening of CD8^+^ T cell epitopes for vaccine candidates, as one may miss out CD8^+^ epitopes that are useful in generating protective CD8^+^ T cells. Besides, evaluations of pathogen-specific CD8^+^ T cell activities in diseases settings should be cautious when IFNγ is the only marker used in the assessments. We believe that our multiplex assay might able to fill these gaps.

## Data Availability Statement

The datasets generated for this study are available on request to the corresponding author.

## Ethics Statement

The studies involving human participants were reviewed and approved by the Human Research Ethics Committee of the University of Hong Kong and Monash Health (HREC/15/MonH/64), Royal Melbourne Hospital (local reference number: 2016/196). The patients/participants provided their written informed consent to participate in this study. The animal study was reviewed and approved by the Committee on the Use of Live Animals in Teaching and Research of The University of Hong Kong; the Institutional Animal Care and Use Committee of the Agency of Science, Technology and Research, Singapore; or the Institutional Animal Care and Use Committee of the University of Iowa.

## Author Contributions

CP, LR, SP, and LP designed experiments. CP, JZ, RC, and ZC performed experiments and data analysis. CP, TN, KK, and LP collected PBMCs for the experiment. CP, RC, LR, KK, SP, and LP wrote the manuscript.

### Conflict of Interest

The authors declare that the research was conducted in the absence of any commercial or financial relationships that could be construed as a potential conflict of interest.
