# Supplementary material for: Multiplex Screening Assay for Identifying Cytotoxic CD8+ T Cell Epitopes
Source: Front Immunol. 2020 Mar 11;11:400. doi: 10.3389/fimmu.2020.00400 (PMC7078160; doi:10.3389/fimmu.2020.00400)
Supplement: Supplementary file 1 [file Table_1.docx]

**Supplementary Tables**

Table S1: Complete list of dye combinations to differentiate 24 groups of splenocytes.

| Groups | CMFDA concentration (µM) | CMTMR concentration (µM) | Deep Red concentration (µM) |
| --- | --- | --- | --- |
| 1 | 5 | - | - |
| 2 | 0.25 | - | - |
| 3 | - | 5 | - |
| 4 | - | 0.5 | - |
| 5 | 5 | 5 | - |
| 6 | 5 | 0.5 | - |
| 7 | 0.25 | 5 | - |
| 8 | 0.25 | 0.5 | - |
| 9 | 5 | - | 2 |
| 10 | 0.25 | - | 2 |
| 11 | - | 5 | 2 |
| 12 | - | 0.5 | 2 |
| 13 | 5 | 5 | 2 |
| 14 | 5 | 0.5 | 2 |
| 15 | 0.25 | 5 | 2 |
| 16 | 0.25 | 0.5 | 2 |
| 17 | 5 | - | 0.1 |
| 18 | 0.25 | - | 0.1 |
| 19 | - | 5 | 0.1 |
| 20 | - | 0.5 | 0.1 |
| 21 | 5 | 5 | 0.1 |
| 22 | 5 | 0.5 | 0.1 |
| 23 | 0.25 | 5 | 0.1 |
| 24 | 0.25 | 0.5 | 0.1 |

Table S2: List of peptides used in the IAV mouse model setup described in figure 2. The combinations of fluorescent dyes for each group and their route of administration are shown.

| Target cell inoculation route | Peptide | Sequence | CMFDA concentration (µM) | CMTMR concentration (µM) | Deep Red concentration (µM) |
| --- | --- | --- | --- | --- | --- |
| Intranasal | DMSO | - | 5 (G++) | - | - |
| Intranasal | CSP280 | SYVPSAEKI | - | 5 (R++) | - |
| Intranasal | NP147 | TYQRTRALV | 0.25 (G+) | - | - |
| Intranasal | HA518 | IYSTVASSL | - | 0.5 (R+) | - |
| Intravenous | DMSO | - | 5 (G++) | - | 2 (Deep Red+) |
| Intravenous | CSP280 | SYVPSAEKI | - | 5 (R++) | 2 (Deep Red+) |
| Intravenous | NP147 | TYQRTRALV | 0.25 (G+) | - | 2 (Deep Red+) |
| Intravenous | HA518 | IYSTVASSL | - | 0.5 (R+) | 2 (Deep Red+) |

Table S3: List of peptides used in the multiplex *in vitro* cytotoxicity assay with human PBMCs in figure 3. These are known HLA-A*11:01 epitopes that were identified from Influenza Research Database. The known HLA-A*11:01 epitopes from Ebola virus were identified from Immune Epitope Database (IEDB).

| Peptide | Sequence | IEDB ID |
| --- | --- | --- |
| DMSO | - |  |
| Ebo 580-588 | RTFSILNRK | 56056 |
| H7N7 PB1 488-497 | GTFEFTSFFY | 22647 |
| H1N1 M2 69-77 | KSMREEYRK | 33447 |
| H1N1 M1 178-187 | RMVLASTTAK | 54953 |
| H1N1 M1 13-21 | SIIPSGPLK | 58567 |
| H3N2 M1 125-134 | ASCMGLIYNR | 144210 |
| H5N1 PA 104-113 | KFLPDLYDYK | 144377 |
| H7N7 PB1 471-480 | KLVGINMSKK | 144383 |
| H1N1 M2 44-53 | RLFFKCIYRR | 144461 |
| H1N1 PB2 322-331 | SFSFGGFTFK | 144475 |
| H3N2 NP 407-416 | SVQPTFSVQR | 144488 |
| H5N1 NP 413-422 | SVQRNLPFER | 144489 |
| H3N2 PB2 690-699 | VLRGFLILGK | 144526 |
| H1N1 M1 239-248 | AYQKRMGVQM | 175777 |
| H1N1 PA 649-658 | LYASPQLEGF | 176343 |
| H1N1 NP 91-99 | KTGGPIYRR | 181219 |
| H1N1 NP 188-198 | TIAMELIRMIK | 566921 |

Table S4: List of peptides used in the multiplex *in vivo* cytotoxicity assay with the vaccination/IAV challenge mouse model in figure 4. Epitope homologues were grouped together and the homologues (in gray) were identified through comparison of the relevant proteome against the proteome of H1N1 PR8 IAV.

| Peptide | Sequence | IEDB ID |
| --- | --- | --- |
| DMSO | - |  |
| CSP280 | SYVPSAEKI | From (Hafalla et al., 2003) |
| NP147 | TYQRTRALV | 67436 |
| HA518 | IYSTVASSL | 29690 |
| FluHA-3 | LRSLVASSG | H3N2 homologue |
| FluNP-1 | ASNENMETM | 4602 |
| FluNP-2 | ASNENMDAM | H3N2 homologue |
| FluNP-3 | ASNENMEAM | H5N1 homologue |
| FluNP-4 | AYERMCNIL | 5757 |
| FluNP-5 | FIKGTKVVPRGKLSTR | H1N1 homologue |
| FluNP-6 | FIRGTKVSPRGKLSTR | 16283 |
| FluNP-7 | FIRGTRVVPRGQLSTR | H5N1 homologue |
| FluNP-8 | FYIQMCTEL | 18406 |
| FluHA-1 | FYRNLLWLT | 18460 |
| FluHA-2 | FFSRLNWLT | H3N2 homologue |
| FluPB1-1 | GYAQTDCVL | 23311 |
| FluNS1-1 | LGLDIETATRAGKQIVERI | 36197 |
| FluHA-4 | LYEKVKSQL | 40746 |
| FluHA-5 | LFEKTRRQL | H3N2 homologue |
| FluHA-6 | LYDKVRLQL | H5N1 homologue |
| FluM2-1 | ICRPIRNEW | H1N1, H3N2 homologue |
| FluM2-2 | VETPTRNEW | 68383 |
| FluPB1F2-1 | VYWKQWLSL | 72193 |
| FluPB1F2-2 | VSWKQWLSL | H3N2 homologue |

Hafalla JCR, Morrot A, Sano G-I, et al. 2003. Early self-regulatory mechanisms control the magnitude of CD8+ T cell responses against liver stages of murine malaria. J. Immunol. 171(2):964–70.

Table S5: List of peptides used in the multiplex *in vivo* cytotoxicity assay with the malaria mouse model in figure 5.

| Peptide | Sequence | IEDB ID |
| --- | --- | --- |
| DMSO | - | - |
| OVA | SIINFEKL | 58560 |
| Pb1 | SQLLNAKYL | 187087 |
| Pb2 | IITDFENL | 233201 |
| A6 | LSGRYNDL | 156544 |
| D5 | WGDEFEKL | 156551 |
| F4 | EIYIFTNI | 156541 |
| F6 | LLPHFSIL | 156543 |
| G4 | YYYDYDKI | 156554 |
| PbT1 | NCYDFNNI | 691649 |
| S20 | VNYSFLYLF | 192762 |
| TRAP | SALLNVDNL | 192629 |
| Kb17 | IVSFSFQNM | 745476 |

Table S6: List of peptides used in the multiplex *in vivo* cytotoxicity assay with the hDPP4-KI MERS-CoV mouse model in figure 6.

| Peptide | Sequence | IEDB ID |
| --- | --- | --- |
| DMSO | - | - |
| MOG | MEVGWYRSPFSRVVHLYRNGK | 113645 |
| S395 | QVYNFKRL | From (28) |
| S434 | ASNCYSSL | From (28) |
| S483 | TVPHNLTTI | From (28) |
| S1165 | IAPVNGYFI | From (28) |
| M64 | ALSIFSAV | From (28) |
| M156 | GACDYDRL | From (28) |
